# PLAG Exerts Anti-Metastatic Effects by Interfering with Neutrophil Elastase/PAR2/EGFR Signaling in A549 Lung Cancer Orthotopic Model

**DOI:** 10.3390/cancers12030560

**Published:** 2020-02-28

**Authors:** Guen Tae Kim, Kyu Woong Hahn, Sun Young Yoon, Ki-Young Sohn, Jae Wha Kim

**Affiliations:** 1 Korea Research Institute of Bioscience and Biotechnology, 125 Kwahak-ro, Daejeon 34141, Korea; kgt121@kribb.re.kr; 2 Department of Biological Sciences, Hannam University, 1646 Daedeok-daero, Daejeon 34054, Korea; 3 ENZYCHEM life sciences, 10F aT Center 27 Gangnam-daero, Seoul 06774, Korea; syyoon@enzychem.com (S.Y.Y.); sky@enzychem.com (K.-Y.S.)

**Keywords:** PLAG, anti-metastasis, tumor infiltrated-neutrophils (TINs), PAR2 degradation, EGFR transactivation

## Abstract

The effectiveness of chemotherapy and radiotherapy to treat lung cancer is limited because of highly metastatic nature. Novel strategies and drugs to attenuate metastatic activity are urgently required. In this study, red fluorescence proteins (RFP)-labeled A549 human lung cancer cells were orthotopically implantation, where they developed primary tumors. Metastasis in brain and intestines were reduced by up to 80% by treatment with 100 mpk 1-palmitoyl-2-linoleoyl-3-acetyl-rac-glycerol (PLAG) compared with that in control mice. PLAG treatment also reduced the migration of the primary tumors. Interestingly, substantial neutrophil infiltration was observed in the tumors in control mice. The neutrophil contribution to A549 cell metastatic activity was examined in in vitro co-culture system. Metastatic activity could be achieved in the A549 cells through epidermal growth factor receptor (EGFR) transactivation mediated by protease activating receptor 2 (PAR2) receptor. Neutrophil elastase secreted from tumor-infiltrating neutrophils stimulated PAR2 and induced EGFR transactivation. However, this transactivation was inhibited by inducing PAR2 degradation following PLAG treatment and metastatic activity was effectively inhibited. PLAG attenuated cancer metastatic activity via modulated PAR2/EGFR transactivation by accelerating PAR2 degradation. These results suggest PLAG as potential therapeutic agent to combat tumor metastasis via regulating the activation signal pathway of PAR2 by tumor infiltrate-neutrophils.

## 1. Introduction

Lung cancer has a much higher mortality rate than other cancers [1,2]. Lung cancer is not easily detected at an early stage and has robust metastatic activity, which contributes to the low survival rate. Metastasized lung cancer is frequently observed in the brain, liver, and intestines, making it difficult to treat with conventional therapies [3,4,5]. The survival rate of patients varies significantly according to the metastasis. The metastatic activity of lung carcinoma is strongly affected by overactivation of growth factors, eventually leading to metastasis and poor prognosis [6,7]. Hence, drug development against metastasis is crucial for the treatment of lung cancer.

Tumor-infiltrating neutrophils (TINs) play vital roles in the abnormal growth and metastasis via activates specific signaling pathways [8,9,10]. Especially, signaling pathways stimulated by elements from TINs such as neutrophil elastase was known to actively induce cancerous process and metastatic activity [11,12]. For this reason, tumor tissues recruited neutrophils into the tissues through various pathways. Tumor tissues recruit neutrophils by releasing chemotactic chemokines [13,14,15,16], and secrete growth factors, causing an abnormal increase in the number of neutrophils [17,18,19]. Recent studies showed that it is possible to inhibit tumor progression by suppressing the recruitment of neutrophils to cancer tissues [20,21]. TINs communicate with cancer cells. In particular, neutrophil elastase released from activated neutrophils stimulates protease activating receptor 2 (PAR2) in cancer, leading to the activation of EGFR via HB-EGF and the induction of uncontrolled progression [22,23,24]. The continuous production of neutrophil elastase by TINs maintains the EGFR activity of cancer through PAR2 stimulation.

The over-activation of EGFR in cancer is a major reason for metastasis. In particular, the overexpression of EGFR found in many lung carcinomas is one of the leading causes of poor prognosis [25,26,27]. EGFR, which can be over-activated through various pathways, regulates the expression of EMT-inducing factors, thereby forming a condition in which cancer can migrate to other tissues [28,29]. Previous studies have investigated cross-activation of EGFR by factors in the surrounding environment is crucial factors to cancer progression [24,30,31]. Especially, PAR2/EGFR over-activation caused by TINs was shown to be an essential part of metastasis. Hence, the blocking of PAR2 activation might be a core device for inhibition of neutrophil-mediated EGFR activation. Furthermore, it might be possible to attenuate neutrophil-mediated tumor metastasis.

The 1-palmitoyl-2-linoleoyl-3-acetyl-rac-glycerol (PLAG) is present in the antlers of the Sika deer and can be synthesized from glycerol, palmitic acid, and linoleic acid [32]. PLAG was recently shown to reduce the gemcitabine-induced neutropenia, oral mucositis and arthritis by modulating neutrophil movement [33,34,35]. There have not yet been any studies to determine if PLAG has the potential to control cancer progression by regulating neutrophil infiltration.

We constructed an animal model using orthotopically implanted A549 human lung cancer and monitored metastatic activity in order to evaluate the efficacy of PLAG for the inhibition of metastasis. We also used in vitro assays to verify the biological activity of PLAG. Our results showed that PLAG effectively inhibits tumor metastasis by blocking EGFR transactivation via prompt PAR2 degradation. PLAG might therefore be utilized as a novel drug to inhibit the metastasis of lung cancer.

## 2. Results

### 2.1. PLAG Inhibited Metastasis of A549 Human Lung Cancer in an Orthotopic Mouse Model.

**Figure 1:**
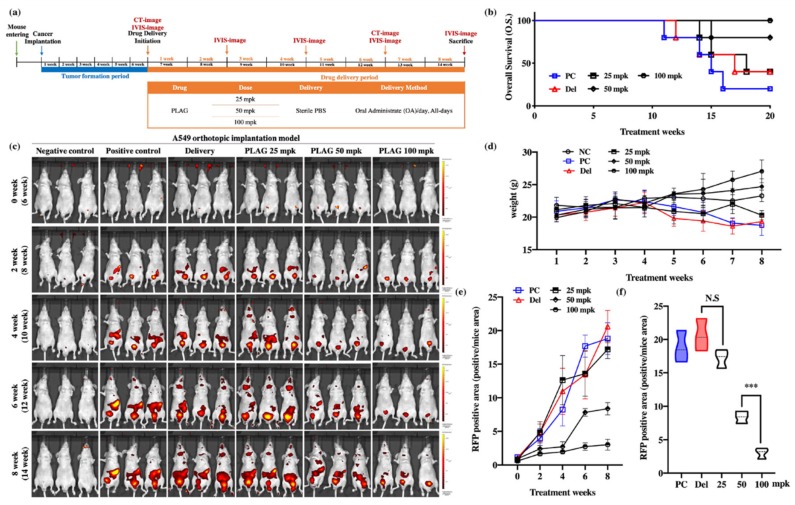
Inhibition of cancer metastasis by 1-palmitoyl-2-linoleoyl-3-acetyl-rac-glycerol (PLAG) in the A549-orthotopically implanted mouse model: (**a**) Experimental design to evaluate the anti-metastatic effect of PLAG in the A549-orthotopically implanted mouse model; (**b**) Analysis of overall survival (OS) among PLAG-treated A549-orthotopically implanted mice over 20 weeks; (**c**) Analysis of tumor metastasis in PLAG-treated mice by in vivo imaging system (IVIS) imaging at 2-week intervals; (**d**) Weight change in PLAG co-treated mice evaluated over 8 weeks; (**e**) Change in RFP-positive areas in each group estimated by Living Image software; (**f**) The RFP-positive areas in each group were compared after 8 weeks. Compared with the positive control group: ***p < 0.001 (each experiment *n* = 5). N.S., not significant. Mean ± SD. NC; negative control, PC; positive control, Del; delivery.

Mice were orthotopically implanted with RFP-labeled A549 human cancer and either left untreated (control) or treated with 25, 50 or 100 mpk PLAG (Figure 1a). At 20 weeks after implantation, all five mice were alive in the PLAG 100 mpk group, four of the five mice were alive in the PLAG 50 mpk group, and one of the five mice was alive in the control group (Figure 1b). Bodyweight gradually decreased in the control group but not in the 50 and 100 mpk PLAG groups (Figure 1d) Metastasized A549 cells were observed in the intestines of the mice 8 weeks after implantation. The PLAG treatment reduced the numbers of metastasized cells in the intestines, inhibiting metastasis by more than 80% in the PLAG 100 mpk group compared with that in the control group (Figure 1c). At 14 weeks after cancer cell implantation, the 50 and 100 mpk PLAG treatments showed an inhibitory effect on the metastasis (Figure 1e,f).

### 2.2. Treatment with 100 mpk PLAG Reduced Metastasis to the Brain and Intestines.

**Figure 2:**
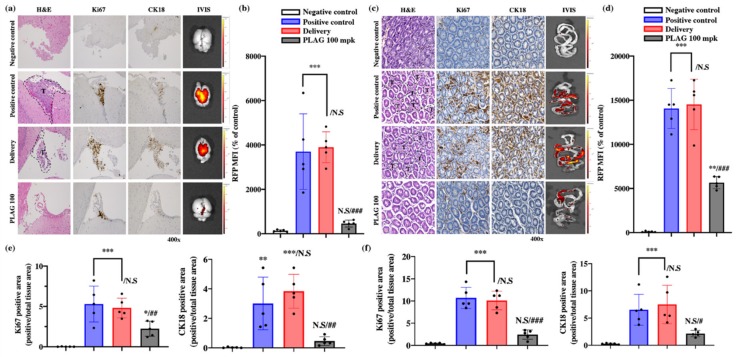
Inhibitory effects of PLAG on tumor metastasis: (**a**) Inhibition of tumor metastasis into the brain in PLAG-treated mice was verified by immunohistochemical staining with anti-Ki67 and anti-CK18 antibodies; (**b**) The RFP-positive MFI value in the brain regions of each group. Compared with the negative control group: *p < 0.05, **p < 0.01, *** *p* < 0.001. N.S., not significant. Compared with the positive control group: ## *p* < 0.01 (each experiment *n* = 5). N.S., not significant. Mean ± SD; (**c**) Inhibition of tumor metastasis into the intestines in PLAG-treated mice was verified by immunohistochemical staining with anti-Ki67 and CK18 antibodies; (**d**) The RFP-positive MFI value in the intestines of each group were calculated. Compared with the negative control group: * *p* < 0.05, ** *p* < 0.01, *** *p* < 0.001. N.S., not significant. Compared with the positive control group: ## *p* < 0.01 (each experiment *n* = 5). N.S., not significant. Mean ± SD; (**e**) The area of cancer metastasis to the brain region was evaluated using human-specific antibodies against Ki67 and CK18. Compared with the negative control group: * *p* < 0.05, ** *p* < 0.01, *** *p* < 0.001. N.S., not significant. Compared with the positive control group: ## *p* < 0.01 (each experiment *n* = 5). N.S., not significant. Mean ± SD; (**f**) The area of cancer metastasis to the gastrointestinal tract was evaluated using human-specific antibodies against Ki67 and CK18. Compared with the negative control group: * *p* < 0.05, ** *p* < 0.01, *** *p* < 0.001. N.S., not significant. Compared with the positive control group: ## *p* < 0.01 (each experiment *n* = 5). N.S., not significant. Mean ± SD.

At 14 weeks after A549 cell implantation, the mice were sacrificed, and the brains and intestines were analyzed by hematoxylin and eosin (H&E) staining, immunohistochemical staining, and IVIS imaging. IVIS imaging of RFP-labeled A549 cells revealed that 100 mpk PLAG treatment inhibited metastasis to the brain and intestines by 93% and 83%, respectively (Figure 2a–d). Labeling with human-specific antibodies to Ki67 and CK18 also revealed metastasis in the brain and intestinal tissues that was markedly reduced by the 100 mpk PLAG treatment (Figure 2e,f).

### 2.3. PLAG Treatment Inhibited the Growth of A549 Human Lung Cancer in Mice.

**Figure 3:**
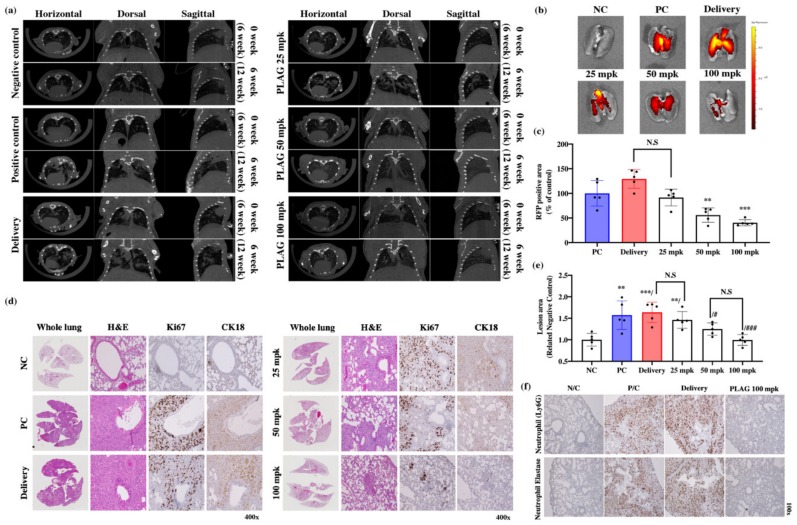
Inhibitory effects of PLAG on A549 human lung cancer cells implanted in mice: (**a**) Orthotopic tumor cell proliferation in mouse lungs determined by CT imaging; (**b**,**c**) Orthotopic tumor cell proliferation in mouse lungs determined by IVIS imaging. Compared with the negative control group: ** *p* < 0.01, *** *p* < 0.001 (each experiment *n* = 5). N.S., not significant. Mean ± SD; (**d**) Evaluation of the main tumor region in PLAG-treated lung tissues after implantation with A549 cells. The volume of alveolar space occupied by tumor cells was measured and compared with that in negative control mice using Image J. Compared with the negative control group: ** *p* < 0.01, *** *p* < 0.001. N.S., not significant. Compared with the positive control group: # *p* < 0.05, ## *p* < 0.01 (each experiment *n* = 5). N.S., not significant. Mean ± SD; (**e**) Tumor growth in PLAG-treated lung tissues evaluated by H&E staining and immunohistochemical staining with anti-Ki67 and anti-CK18 antibodies 14 weeks after implantation; (**f**) Labeling with anti-neutrophil and neutrophil elastase antibody showed that PLAG treatment reduced the numbers of tumor-infiltrating neutrophils in the orthotopic lung tumors.

CT scans revealed that treatment with PLAG reduced A549 tumor growth in the mouse lungs compared with control mice at 12 weeks after the implantation (Figure 3a). Those observations were confirmed by IVIS imaging of lung tissues isolated in sacrificed mice (Figure 3b). The 100 mpk PLAG treatment dramatically reduced the RFP-positive area in the lung tissue (Figure 3c). Reconstituted whole-lung images with H&E staining revealed that the alveolar tissues were filled with growing A549 cells in the positive control mice the and delivery mice, whereas PLAG treatment reduced the numbers of cancer cells in the alveolar tissues. The lesion areas in the whole lungs were calculated using the intensity of stained condensation (Figure 3d,e). The presence of growing A549 cells in the mouse lungs was verified by immunostaining with anti-Ki67 and CK18 antibodies. The lung tissues from both control groups showed strong immunostaining, whereas those from the PLAG-treated mice showed much weaker immunostaining (Figure 3e).

### 2.4. The Metastatic Properties of A549 Cells were Promoted by the Tumor-Infiltrating Neutrophil Environment and Attenuated by PLAG. 

Recent studies have shown that TINs play a crucial role in tumor proliferation and tumor metastasis [21,36,37]. Infiltrating neutrophils in the primary tumors were identified by immunostaining with anti-Ly6G antibody. TINs were abundant in the lung tissues of the control groups (Figure 3f). Based on this result, we aimed to verify the regulatory effect of PLAG on the metastatic activity of cancer cells by TINs using *in vitro* co-culture system.

**Figure 4:**
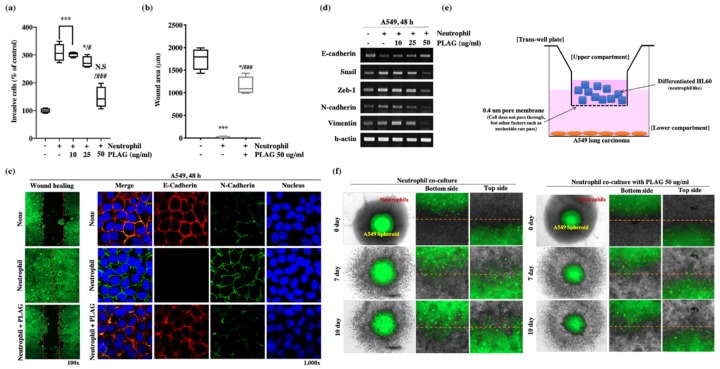
Inhibitory effect of PLAG on metastasis in the tumor-infiltrating neutrophil microenvironment: (**a**) PLAG inhibited the invasive activity of A549 cells in the presence neutrophil. Compared with negative controls: * *p* < 0.05, *** *p* < 0.001. N.S., not significant. Compared with positive controls: # *p* < 0.05, ### *p* < 0.001 (each experiment *n* = 5). Mean ± SD; (**b**,**c**) Inhibitory effect of PLAG on cancer cell mobility was evaluated and analyzed by wound healing assay. Compared with negative control; * *p* < 0.05, *** *p* < 0.001. Compared with the positive control group: ### *p* < 0.01 (each experiment *n* = 5). Mean ± SD. Modulation of E-cadherin and N-cadherin by PLAG in the presence of neutrophil stimulation in A549 cells observed by confocal microscopy; (**d**) Modulation of EMT-related gene expression in PLAG and neutrophil co-treated A549 cells was evaluated by RT-PCR; (**e**) A diagram showing the way that A549 lung cancer cells are activated by neutrophils via indirect contact between cancer cells and neutrophils; (**f**) The inhibitory effect of PLAG in the spheroid invasion assay of A549 cells was calculated on the basis of cells scattering, which is mediated by direct contact with neutrophils.

The promotion of metastatic tumor characteristics by neutrophils was examined using a co-culture system employing trans-well plates (Figure 4e). The porous membrane separating the two chambers of the co-culture system allowed the transfer of intercellular signaling molecules such as cytokines and neutrophil elastase. The upper chamber contained A549, and the bottom chamber contained differentiated HL60. A549 cells moved from the top chamber to the bottom after exposure to the neutrophil; however, treatment with PLAG abolished that invasive activity (Figure 4a). In a wound healing assay, the healing of the simulated wound by A549 cells was enhanced by the presence of neutrophil; however, treatment with PLAG abolished that mobility of the cancer cells (Figure 4c,e). The migration and invasive activity of cancer cells are mainly dependent on the EMT [38]. The modification of EMT-related molecules in A549 cells co-cultured with neutrophils was evaluated by RT-PCR. E-cadherin levels were reduced in the neutrophil-stimulation, whereas the levels of EMT markers were increased. PLAG treatment returned the EMT-related molecules to normal levels (Figure 4c,d). Spheroid assays were conducted to evaluate the promotion of A549 progression as a result of direct contact between GFP-labeled A549 cells and neutrophils. Invasive characters, scattering, and outgrowth of A549 cells were observed after 7 days and 10 days of co-culture, but those abnormal activities were not observed when the PLAG treatment (Figure 4f). Together, these results showed that TINs play a pivotal role in enhancing the metastasis, whereas PLAG strongly attenuates the neutrophil-induced promotion of metastatic tumor activity.

### 2.5. EGFR Transactivation for Metastatic Activity was Mediated by the PAR2 Receptor in the Presence of Neutrophils.

**Figure 5:**
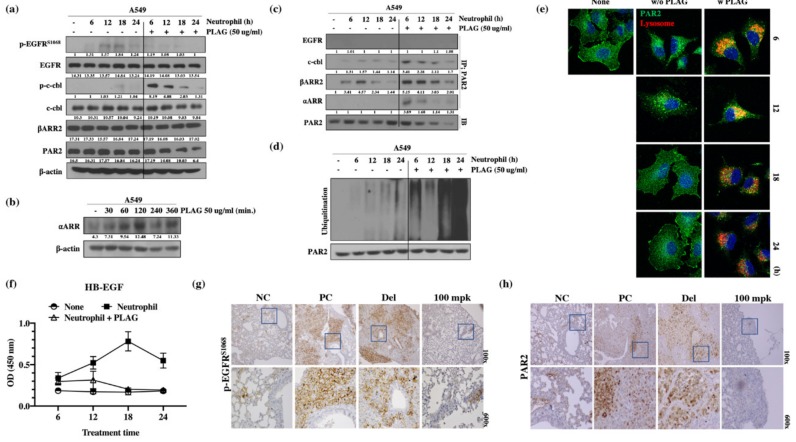
PLAG inhibition of EGFR transactivation mediated by the neutrophil elastase/PAR2/βARR2 signaling pathway in A549 cells: (**a**) Inhibition of the EGFR signaling pathway and PAR2 degradation by PLAG treatment in neutrophil-stimulated A549 cells evaluated by Western blot; (**b**) Induction of ⍺ARR expression by PLAG treatment was confirmed by Western blotting; (**c**) Changes in complex formation between endocytosis-related proteins and PAR2 degradation following PLAG treatment; (**d**) Validation of the PAR2 ubiquitination-inducing effect of PLAG in neutrophil-activated A549 cells; (**e**) Lysosomal PAR2 degradation in PLAG-treated cells was verified by confocal microscopy; (**f**) Changes in HB-EGF secretion following PLAG treatment and neutrophil stimulation in A549 cells; (**g**) Phosphorylation of EGFR; (**h**) and expression of PAR2 in the lung tissues of A549-orthotopically implanted mice were prominently reduced by PLAG treatment.

To verify the enhancement of metastatic activity by the TINs, the PAR2 signaling pathway and EGFR activity of A549 cells mediated by neutrophil elastase were calculated. Western blot analysis for EGFR activity showed that the amount of phosphorylated EGFR was increased in A549 cells after neutrophil stimulation for 12 h, whereas co-treatment with PLAG abolished the neutrophil-induced enhancement of EGFR activity. In addition, c-Cbl phosphorylation and PAR2 degradation were observed in A549 cells after 18 h and 24 h of neutrophil stimulation (Figure 5a). PAR signal-mediated neutrophil elastase is crucial for EGFR trans-activity for metastasis [24,31]. The PAR2 or GPCR, activated by neutrophil elastase was assembled with β-arrestin-2 (βARR2) and ubiquitin ligase in some cases and subjected to intracellular trafficking as a form of endocytosis [39]. Accelerated degradation of PAR2 in the PLAG co-treated A549 cells was verified by immunoprecipitation assay with anti-PAR2 antibody. For intracellular trafficking of PAR2, assembled βARR2 were detected in neutrophil-stimulated A549 cells, but 50 mpk PLAG co-treatment accelerated PAR2 degradation through prompt complex formation with c-Cbl and βARR2 (Figure 5c). Increased ubiquitination and degradation of PAR2 in the PLAG co-treated cells was verified by ubiquitination assay with PAR2 antibody and by confocal microscopy (Figure 5d,e). HB-EGF secretion is crucial signaling pathway for EGFR transactivation mediated by GPCR [30,40]. Increased HB-EGF secretion was observed in the neutrophil-treated A549 cells, but the HB-EGF secretion was completely inhibited in PLAG co-treated cells (Figure 5f). Those results indicate that EGFR transactivation was induced in the presence of neutrophil stimulation via the PAR2 signaling pathway, and the transactivation was effectively inhibited in PLAG co-treated cells via prompt PAR2 degradation. Similar to the pattern of EGFR activation and PAR2 degradation observed in vivo, EGFR phosphorylation and PAR2 expression were modulated by PLAG in the lung tissues of tumor-implanted mice (Figure 5g,h).

### 2.6. Neutrophil-Induced Metastatic Activity of A549 Cells was Dependent on PAR2 and β-Arrestin-2.

**Figure 6:**
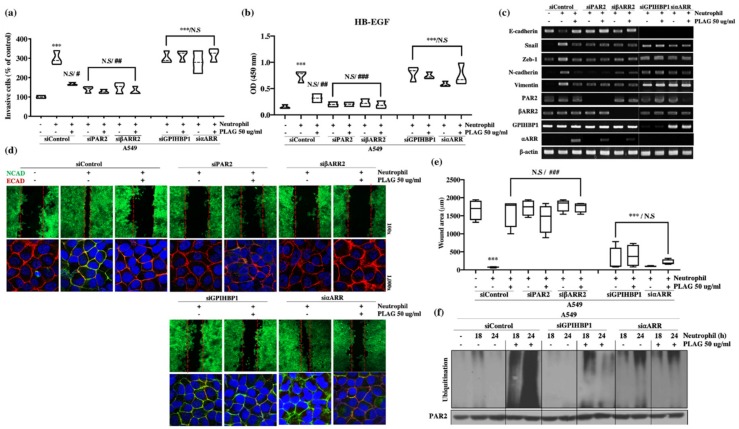
PLAG inhibition of neutrophil-induced invasive activity in A549 cells verified by silencing of PAR2/βARR2 and GPIHBP1/⍺ARR cascade: (**a**) Neutrophil-induced enhancement of invasive activity in A549 cells was not observed in PAR2-silenced (siPAR2) or β-arrestin 2-silenced (siβARR2) cells. The inhibitory effect of PLAG on the enhancement of invasive activity in A549 cells disappeared in GPIHBP1-silenced (siGPIHBP1) and ⍺-arrestin-silenced (si⍺ARR) cells. Compared with the negative control group: ***p < 0.001. N.S., not significant. Compared with the positive control group: # *p* < 0.05, ## *p* < 0.01 (each experiment *n* = 5). N.S., not significant. Mean ± SD; (**b**) Enhanced HB-EGF secretion in neutrophil-stimulated A549 cells was not observed in siPAR2 cells or siβARR2 cells. The inhibitory effect of PLAG on the enhancement of HB-EGF secretion in A549 cells disappeared in siGPIHBP1 and si⍺ARR. Compared with the negative control group: * *p* < 0.05, *** *p* < 0.001. N.S., not significant. Compared with the positive control group: ## *p* < 0.01 (each experiment *n* = 5). N.S., not significant. Mean ± SD; (**c**) Modulation of EMT-related gene expression in neutrophil-stimulated A549 cells with or without PLAG treatment in the target proteins silenced; (**d**,**e**) Cancer mobility in neutrophil-stimulated A549 cells with or without PLAG treatment in the target proteins silenced. Compared with the negative control group: *** *p* < 0.001. N.S., not significant. Compared with the positive control group: ### *p* < 0.001 (each experiment *n* = 5). N.S., not significant. Mean ± SD; (**f**) PAR2 ubiquitination changes with or without PLAG treatment in the target proteins silenced.

The main factors in the neutrophil-induced stimulation of metastatic activity were verified by gene silencing of PAR2 and βARR2. Invasiveness, heparin binding-epidermal growth factor (HB-EGF) secretion, modulated expression of EMT-related genes, and wound healing activity were abolished entirely in the PAR2 or βARR2-silenced (Figure 6a–e). Those results suggest that the metastatic activity induced by neutrophils is dependent on PAR2 for recognition of neutrophil elastase and βARR2 for modulation of EMT-related molecules through PAR2 signaling. Activated PAR2 undergoes intracellular trafficking via the βARR2 complex and thus modulates EMT-related gene expression via EGFR transactivation. 

### 2.7. The Inhibitory Effect of PLAG on Neutrophil-Induced Metastatic Activity in A549 Cells was Dependent on GPIHBP1 and ⍺-Arrestin.

The blocking of metastatic activity by PLAG in A549 cells was verified by gene silencing of the vesicle-recognizing receptor GPIHBP1. PLAG, an acetylated diacylglycerol, forms a vesicle in hydrophilic culture media under slight agitation. The PLAG vesicle is trapped and recognized by GPIHBP1 similarly to the way chylomicrons are recognized and trapped in peripheral tissues [41]. The modified vesicle surface with acetylation is recognized by its cognate cell-surface receptor and thus induces cellular signaling. PLAG treatment induced ⍺-arrestin (⍺ARR) in A549 cells (Figure 5b). Invasiveness, HB-EFG secretion, expression of EMT-related genes, and wound healing activity inhibition effects were significantly reduced in GPIHBP1-silenced A549 cells (Figure 6a–e). The increased ⍺ARR level boosted GPCR intracellular trafficking, the formation of ⍺-arrestin complex including ubiquitin ligase, and eventually the degradation of GPCR [42]. Increased PAR2 degradation was frequently detected in A549 cells with PLAG-induced elevation of ⍺ARR levels. Together, the results suggest that PLAG induced ⍺ARR, and the degradation of neutrophil-stimulated PAR2 was achieved via promotion of the ⍺ARR complex including ubiquitin ligase.

PLAG forms vesicles in cell culture media and a major component of the micelle membrane, which is captured by GPIHBP1, a vesicle-capturing ligand of cancer cells. (P1) The captured PLAG itself stimulates cells and induces ⍺ARR expression. (P2 and 3) Increased ⍺ARR is swiftly assembled with ubiquitin ligase. (P4) Prompt degradation of PAR2 (desensitization). In conclusion, inhibition of metastatic activity by PLAG is mainly dependent on interference with the PAR2/EGFR/EMT signal cascade caused by prompt PAR2 degradation in cancer cells in the presence of neutrophils.

## 3. Discussion

The arbitrary modulation of metastatic behavior in tumor might be the best way to improve mortality rates among patients. Recent studies showed that neutrophil infiltration in primary tumors induces aggressive behaviors such as metastasis and abnormal proliferation [37,43,44]. Cancerous tissues often express chemokines for the recruitment of neutrophils. In particular, the expression of chemokines such as CXCLs leads to rapid recruitment of neutrophils. Neutrophils infiltrated in tumor tissue secrete elements such as neutrophil elastase and it induce tumor tissue overactivity. Neutrophils also promote metastasis by releasing cytokines and associating with circulating tumor cells [45,46,47]. In in vivo cancer models, the inhibition of CXCR2 reduces neutrophil recruitment to tumors and increases the efficacy of chemotherapy [44,48,49]. The progression inhibitors of tumor tissues targeting TINs so just focused on inhibiting TINs recruitment. However, the mechanism and substances that can regulate the activity of tumor by already infiltrated neutrophils have not been studied yet.

In this study, we have identified an effect and mechanism of PLAG that inhibits the metastatic activity of tumor by infiltrated neutrophils. Our results revealed that PLAG treatment nearly abolished metastasis into the brain and intestines (Figure 1 and Figure 2). In in vitro assays confirmed that migration and expression of EMT-related molecules were activated in presence of neutrophils and attenuated by PLAG (Figure 4). Those results suggest that PLAG effectively reversed the neutrophil-induced aggressive properties of lung cancer and might therefore be useful as an anti-metastatic agent.

Neutrophil elastase released from activated neutrophils is one of the major components that promote tumor metastasis [11,12,50]. The endogenous neutrophil elastase inhibitor is involved in inflammation-mediated tumor progression [51]. Our results confirmed that the metastatic properties induced by neutrophils and confirmed by invasion assays and wound healing assays were abolished in PAR2-silenced cells. Furthermore, the neutrophil-induced modulation of EMT-associated molecules was also absent in PAR2-silenced cells (Figure 6).

**Figure 7:**
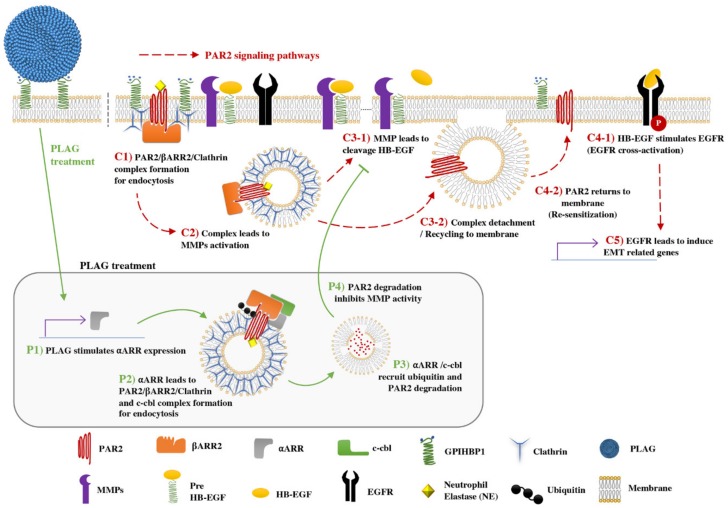
PAR2/EGFR-mediated expression of EMT-related genes; PLAG accelerates PAR2 degradation through ⍺-arrestin expression and assembly of ubiquitin ligase: C1) Tumor-infiltrating neutrophils secrete neutrophil elastase, which stimulates the PAR2 receptor. C2) Neutrophil elastase-stimulated PAR2 starts intracellular trafficking via assembly of βARR2 and clathrin (internalization complex). Internalized PAR2 leads to MMPs activation. C3-1) MMP cleavage of pre-HB-EGF and release of HB-EGF. C3-2) Internalization complex was detached from PAR2 and C4-2) PAR2 returns to the plasma membrane (re-sensitization). C4-1) Released HB-EGF stimulates EGFR and C5) EMT-related gene expression. EGFR transactivation and signaling induces several genes involved in EMT of cancer cells. Major metastatic activity of cancer cells can be achieved through modulation of EMT-related gene expression.

High levels of ⍺ARR transcripts were observed in A549 cells within 60 min of stimulation with PLAG (Figure 5b). In addition, neutrophil elastase-stimulated PAR2 was assembled with ⍺, βARR, c-Cbl (Figure 5c). Successive ubiquitination and degradation of c-Cbl-associated PAR2 were detected in PLAG-treated cells and primary lung tumors (Figure 5d,h). Those results suggest that the main role of PLAG in the retardation of tumor metastasis is the attenuation of EGFR transactivation via PAR2 degradation (Figure 7). The anti-metastatic effects of PLAG mediated by PAR2 degradation were validated in GPIHBP1-silenced or ⍺ARR-silenced cells (Figure 6). Overall, the results confirmed that PLAG effectively inhibited the metastasis of A549 lung cancer cells into the brain and intestines in the A549 orthotopic mouse model. The anti-metastasis effect of PLAG might be achieved by the disruption of PAR2 signaling, which mediates tumor cell activation in the presence of neutrophil elastase.

PLAG has been studied in various inflammatory diseases including chemotherapy-induced neutropenia (CIN), chemo/radiation-induced oral mucositis (CRIOM), and rheumatoid arthritis (RA) [33,34,35]. Our results suggest that PLAG as a novel candidate molecule for the inhibition or retardation of tumor metastasis. Effective blocking of metastasis-promoting signals via PAR2 degradation by PLAG treatment offers a potential pathway for novel drug development. Taken together, our results suggest that PLAG might be used as a new anti-metastasis agent via regulating the activation signal pathway by neutrophils infiltrating cancer tissue.

## 4. Materials and Methods

### 4.1. Cell Culture

A549 and HL60 cells were obtained from the American Type Culture Collection (ATCC, Manassas, MD, USA). Both types of cells were grown in Dulbecco’s modified Eagle medium (DMEM; WelGENE, Seoul, Korea) containing 10% fetal bovine serum (HyClone, Waltham, MA, USA) and 1% antibiotics (100 mg/L streptomycin, 100 U/mL penicillin) at 37 °C in a 5% CO_2_ atmosphere. To induce HL60 cells to differentiate into neutrophil-like cells, the cells were grown for 5 days in the presence of 10% DMSO.

### 4.2. Invasion Assay to Measure the Migration Activity of Cancer Cells

Quantitative cell invasion assays were performed using a modified Boyden chamber (Costar-Corning, NY, USA) on Matrigel-coated 24-well plates containing polycarbonate membrane inserts with 8.0 μm pores. The lower chamber was filled with neutrophils in complete medium. A549 cells (5 × 10^4^ cells/mL) in 1% serum medium were added to the upper chamber with or without PLAG. The cells were allowed to invade for 24 h at 37 °C. Non-invasive cells were removed from the upper surface of the membrane by scraping with a cotton swab, and the numbers of cells that migrated across the membrane to the lower chamber were calculated by methylthiazolyldiphenyl-tetrazolium bromide(MTT) assay.

### 4.3. Co-Culture System for Indirect Contact using Separated Chambers 

A549 cells were seeded in 6-well or 24-well plates. The pre-culture media was then removed, and 1.5 mL (for 6-well plates) or 0.5 mL (for 24-well plates) fresh media was added to each well. A polycarbonate membrane insert with 0.4-μm pores was then inserted into each well to create a modified Boyden chamber (SPL Lifescience, Seoul, Korea). Differentiated HL60 cells suspended medium were added to the upper chamber to stimulate the A549 cells. PLAG was added to the lower chamber 1 h before stimulation with the differentiated HL60 cells. The co-culture ratio between A549 and dHL60 cells was combined at A549:dHL60 = 1:5.

### 4.4. Immunofluorescence Staining and Wound Healing Assay

A549 cells were seeded at a density of 2.5 × 10^6^ cells/mL on 12-well plates with a cover glass and grown to 100% confluence. A wound was then made on the cell monolayer on the center of the well. The cultures were then treated with PLAG and neutrophil stimulation at 37 °C in a 5% CO_2_ atmosphere. The cells were then fixed with 3.7% formaldehyde for 20 min and permeabilized with 0.2% Triton X-100 for 20 min to stain the actin fibers. To stain for NCAD (Santacruz, sc-1502, 1:1000, Dallas, TX, USA) and ECAD (Santacruz, sc-7870, 1:1000, Dallas, TX, USA), the cells were fixed with 3.7% formaldehyde for 20 min, washed with PBST twice, and reacted with specific antibodies overnight at 4 °C. The cells were then washed with PBST twice and reacted with secondary antibodies. Fluorescence was detected by confocal microscopy (Carl Zeiss, Thornwood, NY, USA). The degree of wound healing was quantified using Image J.

### 4.5. Spheroid-Formation Assay to Examine Cancer Cell Scattering

A total of 5 × 10^3^ GFP-labeled A549 cells were plated onto an ultra-low binding clear U-bottom plate with 100 μL growth medium. The cells in the middle of the bottom of the plate were centrifuged and incubated for 5 days. Once spheroids were established, 70 μL of the culture medium was removed and replaced with 70 μL Matrigel containing neutrophils with or without PLAG. After incubation for 15 min, the Matrigel was hardened. Then, 50 μL growth medium was added and incubated for a predetermined time. The degree of spheroid scattering was determined and quantified using the Image Xpress system (Molecular Device Corporation, CA, USA) for GFP fluorescence detection.

### 4.6. Analysis of Protein Degradation by Ubiquitination Assay

A549 cells were treated with PLAG and stimulated with neutrophils for various times after 10 μM MG132 pre-treatment for 2 h at 37 °C in a 5% CO_2_ atmosphere. The cells were then lysed using ice-cold immunoprecipitation lysis buffer (25 mM Tris-HCl pH 7.4, 150 mM NaCl, 1% NP-40, 1 mM EDTA, 5% glycerol). The extracted proteins were incubated with Surebeads Protein G-specific antibody-bound magnetic beads (Bio-Rad, CA, USA). The beads were then washed with PBST. Ubiquitination of PAR2 was eluted in 1× non-reducing sample butter and analyzed by Western blot.

### 4.7. Analysis of Secreted Proteins by ELISA

The levels of HB-EGF secretion in the cell supernatants or mouse plasma were analyzed by factor-specific ELISA according to the manufacturer’s protocol (R&D Systems, MN, USA). The absorbance was measured at 450 nm using an EMax Endpoint ELISA microplate reader (Molecular Devices Corporation, CA, USA).

### 4.8. Examination by Confocal Microscopy for Lysosome Co-Localization

A549 cells were seeded on a 12-well plate with a cover glass and incubated to 60% confluence. The cells were then treated with PLAG and neutrophil stimulation for various times at 37 °C in a 5% CO_2_ atmosphere. The treated cells were fixed with 3.7% formaldehyde for 20 min, permeabilized with 0.2% Triton X-100 (Sigma Aldrich, St. Louis, MO, USA) for 20 min, washed with PBST twice, and reacted with PAR2 antibody (Santacruz, sc-13504, 1:1000, Dallas, TX, USA) overnight at 4 °C. The cells were then washed with PBST twice and reacted with secondary antibody. Fluorescence was detected by confocal microscopy (Carl Zeiss, Thornwood, NY, USA)

### 4.9. Analysis of Protein Assemblies by Immunoprecipitation

A549 cells were treated with PLAG and stimulated with neutrophils for various times at 37 °C in a 5% CO_2_ atmosphere. The cells were then lysed in ice-cold immunoprecipitation lysis buffer (25 mM Tris-HCl pH 7.4, 150 mM NaCl, 1% NP-40, 1 mM EDTA, 5% glycerol). The extracted proteins were incubated with Surebeads Protein G-specific antibody-bound magnetic beads (Bio-Rad, CA, USA). The beads were then washed with PBST, and target proteins were eluted in 1× sample buffer and analyzed by western blot.

### 4.10. Analysis of Expressed Transcripts using Quantitative PCR

Total RNA was extracted using RiboEx (GeneAll Biotechnology, Seoul, Korea) according to the manufacturer’s instructions. cDNA was generated using the ReverseAids cDNA synthesis kit (Thermo Scientific, Waltham, MA, USA) according to the manufacturer’s instructions. RT-PCR was performed with the following temperature profile: pre-denaturation for 10 min at 95 °C; 35 cycles of 95 °C for 30 s, annealing temperature for 30 s, and 72 °C for 30 s; and a final exposure to 72 °C for 10 min.

### 4.11. Gene Silencing by Small Interfering RNA (siRNA)

siRNA was purchased from Santa Cruz Biotechnology (Dallas, TX, USA). For transient transfection, cells were washed twice with PBS and resuspended in transfection buffer (Lonza, Basel, Switzerland) with siRNA. The cell and siRNA mixtures were placed in a Nucleocuvette and electroporated using a 4D-nucleofector (Lonza, Basel, Switzerland). After transfection, the cells were incubated in differentiation medium for 72 h and treated with PLAG and neutrophil stimulation for various times.

### 4.12. Lung Cancer Orthotopic Implantation Model

Male Balb/c nu/nu mice aged 5 weeks old were obtained from NARA biotech (Yong-in, South Korea) and housed in sterile filter-topped cages. The animals (*n* = 5 for each treatment group) were anesthetized using isoflurane and put in a position of left lateral decubitus. A total of 2 × 10^6^ RFP-labeled A549 cells in a solution containing 20 µL culture medium and 20 µL Matrigel (BD Biosciences, NJ, USA) were directly injected through the intercostal space into the lung to a depth of 3 mm using a 29-G needle permanently attached to a 0.5-mL insulin syringe (Becton Dickinson, NJ, USA). The mice were then allowed to rest on a heating carpet until fully recovered. Starting 6 weeks after implantation of cells, the mice were given daily oral doses of 25, 50 or 100 mpk PLAG (*n* = 5 mice per group). A control group (*n* = 5 mice) was left untreated. Metastasis of the lung cancer cells to other tissues was detected by IVIS (PerkinElmer, MA, USA) every 2 weeks after implantation and by computed tomography (CT) at 6 and 12 weeks after implantation. The animals were sacrificed 14 weeks after implantation and perfused with PBS. The lung tissue and metastatic tissue regions were extracted and fixed with 10% formaldehyde. Hematoxylin and eosin (H&E) and immunohistochemical staining was performed on the tissue sections to survey the tissue morphology. All animal experiments were approved by the IACUC, Korea Research Institute of Bioscience & Biotechnology (approval number: KRIBB-AEC-18200). 

### 4.13. Overall Survival Analysis

Cancer implantation and PLAG treatment were performed in the same manner as above. We plotted Kaplan-Meier survival curves 20 weeks after cancer implantation. This treatment was performed until the final observation week excluding death or moribundity. All animal experiments were approved by the IACUC, Korea Research Institute of Bioscience & Biotechnology (approval number: KRIBB-AEC-18127).

### 4.14. Tumor Mass Scan by Computed Tomography 

Before the CT scan, the mice were anesthetized with isoflurane until complete relaxation. Then, the isoflurane concentration was reduced to 1% and maintained at that level during the rest of the experiment. While intubated, all animals were scanned with a Polaris G90 X-ray micro-CT (NANOfocusray, Seoul, Korea) with a source voltage of 80 kVp and a current of 75 μA. Six hundred projections were acquired with an exposure time of 500 ms per projection. The average scan time was 20 min. All images were analyzed using a Horos DICOM program.

### 4.15. Immunohistochemistry Staining

Tissue specimens from the mice were fixed in 10% formaldehyde, embedded in paraffin, and sectioned into 5 µm slices. The sections were treated with 3% H_2_O_2_ for 10 min to block endogenous peroxidase activity and then blocked with bovine serum albumin. Then, the sections were washed in PBS and incubated with PAR2 (Santacruz, sc-13504, 1:100) and p-EGFR (Santacruz, sc-81488, 1:100), Ly6G (Abcam, ab-25377, 1:100), Neutrophil elastase (Abcam, ab-68672, 1:100) overnight at 4 °C. Negative controls were incubated with the primary normal serum IgG for the species from which the primary antibody was obtained.

### 4.16. Hematoxylin and Eosin Staining

Tissue specimens from the mice were fixed in 10% formaldehyde, embedded in paraffin, and sectioned into 5-µm slices. The sections were stained with hematoxylin (Dako, Santa Clara, CA, USA) for 10 min and rinsed in Scott’s tap water (2% NaHCO_3_ in water). Then, the sections were washed in 100% EtOH and stained with eosin (Dako, Santa Clara, CA, USA) for 5 min. Staining of whole-lung samples was determined using the Image Xpress detection system (Molecular Device Corporation, CA, USA). A total of 168 pictures (12 × 14) were taken at 10× magnification and stitched together into a single image. The lesion areas in the lung alveoli were analyzed using Image J. 

### 4.17. Statistics

The data were analyzed using one-way ANOVA (Prism 8, GraphPad Software, La Jolla, CA, USA). *p* < 0.05 was considered statistically significant.

## 5. Conclusions

The various researchers have tried to suppress the metastasis of lung cancer using various substances. However, many substances could not effectively inhibit metastasis, or even if they inhibited, they could not be used because of their high human toxicity.

Through the previous tests, we have found that the treatment of PLAG is harmless to humans. In addition, by effectively regulating immune function, several disease alleviation effects were verified. In addition to these functions, we present objective data in this paper that PLAG treatment can effectively suppress abnormal metastasis of lung cancer cells. In particular, lung cancer metastasis model was used to prove that inhibition of PLAG metastasis is very effective objectively. A detailed description of the phenotype and the mechanism of action of PLAG is also presented. Considering the characteristics of PLAG without human toxicity, our results suggest that PLAG might play a significant role in the treatment of lung cancer.
